# Impacts of Anti-dsDNA Antibody on In Vitro Fertilization-Embryo Transfer and Frozen-Thawed Embryo Transfer

**DOI:** 10.1155/2017/8596181

**Published:** 2017-04-28

**Authors:** Jiao Fan, Yiping Zhong, Cuina Chen

**Affiliations:** Reproductive Medicine Center for The First Affiliated Hospital of Sun Yat-sen University, Key Laboratory of Reproductive Medicine of Guangdong Province, Guangzhou 510080, China

## Abstract

Our purpose is to explore whether anti-dsDNA antibody, which was demonstrated to enter living cells and induced apoptosis, could adversely affect reproductive outcomes. A total of 259 women receiving the in vitro fertilization-embryo transfer (IVF) cycle were enrolled in this study, including 52 women with positive ANA and anti-dsDNA (ANA+/anti-dsDNA+ group), 86 women with positive ANA and negative anti-dsDNA (ANA+/anti-dsDNA− group), and 121 women with negative ANA and anti-dsDNA (ANA−/anti-dsDNA− group). 136 nonpregnant women among 259 patients in the IVF-ET cycle were enrolled in the hormone replacement therapy frozen-thawed embryo transfer (HRT-TET) cycle. We compared basic characters and IVF outcomes among three groups in fresh embryo transfer and frozen-thawed embryo transfer cycle, respectively. The number of retrieved oocytes, available embryos, and high-quality embryos in the ANA+/anti-dsDNA+ group was lower than those in the other two groups in the fresh embryo transfer cycle. The rates of fertilization, implantation, and clinical pregnancy in the ANA+/anti-dsDNA+ group were the lowest, while the early miscarriage rate was the highest in the ANA+/anti-dsDNA+ group both in the fresh embryo transfer cycle and in the frozen-thawed embryo transfer cycle. Our data suggested that anti-dsDNA antibody may be the essential marker for defective oocytes or embryos in infertile women with any type of ANA.

## 1. Introduction

Antinuclear antibodies (ANAs) were related to infertility, decline of oocyte quality, impairment of embryo development, recurrent spontaneous abortion, and IVF failure [1–4]. ANAs were a large group of autoantibodies targeting the entire cell including DNA, RNA, proteins, and/or their complexes. It is unknown which kinds of ANA were involved in poor reproductive outcomes. As a serological marker for diagnosis of systemic lupus erythematosus (SLE), anti-dsDNA antibody played a crucial role in the pathogenesis of lupus nephritis [5, 6]. There were increasing literature which showed that anti-DNA antibody could penetrate into living cells and interact with their intracellular target [7–13]. These studies from several laboratories contradicted prevailing immunologic dogma that cell interiors were inaccessible to antibodies. Anti-dsDNA antibody could be detected in cellular internal and deposit in the kidney and other organs when anti-dsDNA antibody was administrated into nonautoimmune mice in vivo trial [7, 14]. Similar findings were observed after coculture anti-dsDNA antibody with cells in vitro trail. Studies showed that anti-dsDNA antibodies were cytotoxic to cells and induced apoptosis [15–19]. It is plausible that anti-dsDNA antibody could lead to abnormal development of oocyte and embryo. Thus, the aim of this present study was to explore the clinical significance of anti-dsDNA antibody in oocyte, fertilization, and embryo implantation after IVF-ET and HRT-TET.

## 2. Materials and Methods

### 2.1. Patients

The first part of this study was to investigate influences of anti-dsDNA on IVF-ET cycle. According to the inclusion criteria, a total of 259 women who presented to the IVF program at the Reproductive Medicine Center, The First Affiliated Hospital of Sun Yat-sen University, from December 2013 to May 2016 were recruited. Recruitment criteria were age < 38 years, basal FSH < 10 IU/l, antral follicle count between 6 and 15, no prior history of ovarian surgery, and no prior history of chemotherapy. The main indications for the detection of ANA and anti-dsDNA included IVF failure (≥2 cycles), recurrent IUI failure (≥3 cycles), and history of spontaneous abortion. For all patients, the anti-dsDNA and ANA were tested before the IVF program by the hospital's clinical laboratory. Infertility diagnoses for all patients were as follows: tubal disease in 66 patients, male infertility in 75 couples, combined male and tubal infertility in 53 couples, endometriosis in 22 patients, ovulatory disorders and other diagnoses in 23 patients, and unexplained infertility in 20 couples. Patients with autoimmune diseases or clinical presentations of autoimmune diseases, such as systemic lupus erythematosus, antiphospholipid syndrome, Sjogren's syndrome, scleroderma, and autoimmune thyroiditis, as well as those positive for any other autoantibodies including anticardiolipin antibody, antithyroid antibody, lupus anticoagulant, and rheumatoid factor were excluded from this study. Patients were divided into three groups according to the antibodies profile. A total of 259 women receiving the in vitro fertilization- (IVF-) embryo transfer cycle were enrolled in this study, including 52 women with positive ANA and anti-dsDNA (ANA+/anti-dsDNA+ group), 86 women with positive ANA and negative anti-dsDNA (ANA+/anti-dsDNA− group), and 121 women with negative ANA and anti-dsDNA (ANA−/anti-dsDNA− group). Impacts of anti-dsDNA on the frozen-thawed embryo transfer cycle were explored in the second part. 136 nonpregnant women among 259 patients in the IVF-ET cycle were enrolled, 32 women with positive ANA and anti-dsDNA (ANA+/anti-dsDNA+ group), 48 women with positive ANA and negative anti-dsDNA (ANA+/anti-dsDNA− group), and 56 women with negative ANA and anti-dsDNA (ANA−/anti-dsDNA− group) were included. All patients enrolled in this study were notified about the study, and all of them signed informed consent forms for publishing. The Medical Ethics Committee of The First Affiliated Hospital of Sun Yat-sen University approved this study.

### 2.2. IVF Program

Long-term pituitary downregulation was performed in all patients. In brief, a long-acting GnRH agonist (0.8 mg, 1.0 mg, or 1.3 mg) was administered subcutaneous in the midluteal phase. When complete pituitary downregulation was observed, gonadotropin was given for controlled ovarian hyperstimulation; the dose of gonadotropin was chosen according to the patient's age, antral follicle count, and basal FSH level. Human chorionic gonadotropin (5000–10,000 IU) was injected IM when at least two follicles > 18 mm or more than two follicles > 17 mm in diameter were present. Oocytes were collected approximately 36 hours after hCG injection and subjected to IVF. At most, three embryos were transferred into the uterus on the third day after oocyte collection. In our center, embryo quality is mainly assessed by a day-3 embryo grading system; a good quality embryo is defined as six to eight even, equally sized blastomeres with <20% fragmentation of blastomeres. High-quality embryo was given priority to choose for transfer, and high-quality embryos were transferred to all patients in the in vitro fertilization-embryo transfer cycle of this study. Fourteen days after ET, the urine and serum hCG levels were measured. Once urine and serum hCG examination showed positive results, the patients received ultrasonography 2 weeks later to determine the presence of clinical pregnancy.

### 2.3. Endometrial Preparation in the Hormone Replacement Therapy Frozen-Thawed Embryo Transfer Cycle

136 nonpregnant patients among 259 patients in the IVF cycle began to take pentanoic acid estradiol orally at the third day of menstrual period after IVF-ET treatment for 3 months or above. Initial dose was selected on the basis of former endometrial conditions detected by ultrasound. Patients need to receive blood tests and ultrasonic inspections at regular intervals to adjust medical dosage according to the outcomes of endometrial thickness and serum level of sex hormones. When endometrial thickness reached 8 mm or above and serum level of estradiol was higher than 100 pg/ml, progesterone (40 mg/d) was intramuscularly injected to translate endometrium into the secretory phase. After the third day, embryos were transplanted at the fourth day of progesterone injection. At most, 3 embryos were allowed to transplant into the uterine cavity. Progesterone supplementation by daily intramuscular injection of 40 mg per day was applied for all patients. Pregnancy was diagnosed by positive blood test for HCG at 14 days after the third day of embryo transfer. Clinical pregnancy was confirmed by the detection of the gestational sac with a fetal heartbeat by transvaginal ultrasound examination after positive HCG test for 2 weeks.

### 2.4. ANA and Anti-dsDNA Detection

All patients detected anti-dsDNA and ANA before the IVF program.

Serum ANAs were detected by the indirect immunofluorescence assay (IFA) on a slide with human epithelial HEp-2 cell line and liver tissue (monkey) substrate (EUROIMMUN, Lübeck, Germany) in dilution ratios of 1 : 100, 1 : 320, and 1 : 1000. ANAs would react with the antigens in the HEp-2 cell substrate, forming antigen antibody complexes bound to the cell nucleus. The slides were prepared following the manufacturer's recommendations and protocol and were evaluated under the fluorescence microscope using ×20 or ×40 objectives. The ANA test was considered positive when the characteristic fluorescent signal was detected in the tissue or cells, with a serum dilution ratio of 1 : 100 (EUROIMMUN). Anti-dsDNA was tested by the indirect immunofluorescence assay (IFA) on a slide with *Crithidia luciliae*. Anti-dsDNA was defined as positive only when fluorescence staining was detected in kinetoplasts of *Crithidia luciliae*. All assays were performed and interpreted according to the manufacturer's protocol.

### 2.5. Data Collection

The basic clinical information was collected including the age, duration of infertility, basal level of FSH and LH, endometrial thickness on the day of HCG injection, and number of embryo transferred and data of outcomes of IVF: number of oocytes retrieved, available embryos, high-quality embryos, fertilization rate (2Pb or 2PN oocytes in each group/total retrieved oocytes in the IVF cycle), clinical pregnancy rate (patients detected with fetal heartbeat in each group/total patients in each group), implantation rate (embryos with fetal heartbeat in each group/total transferred embryos), and early miscarriage rate (patients with pregnancy loss within 12 weeks of clinical pregnancy in each group/total patients with clinical pregnancy).

### 2.6. Statistics

Statistical analysis was done with SPSS version 20.0 statistic software package. The Kruskal-Wallis test was performed to analyze differences in quantitative data among the three groups, and the Mann-Whitney *U* test was used to analyze differences between any two groups if difference among the three groups was statistically significant. A value of *P* < 0.05 was considered to indicate statistical significance among the three groups, and a *P* value of <0.0167 was used to indicate statistical significance between any two groups. A chi-squared test and partition of chi-squared test were used to compare qualitative data; likewise, *P* < 0.05 was considered to be statistically significant among the three groups, and *P* < 0.0167 was used to indicate statistical significance between two groups. Fisher's exact test was used as appropriate.

## 3. Results

### 3.1. Background of Patients' Characteristics

Either in the fresh embryo transfer cycle or the in frozen-thawed embryo transfer cycle did not exist marked differences in the age, AFC, duration of infertility, bFSH, and bLH among the three groups (Tables 1 and 3).

### 3.2. Controlled Ovarian Stimulation and IVF-ET Outcome

Statistical analysis showed there were no significant differences in the days of ovarian stimulation, total Gn dose, E2 level on the day of HCG treatment, and the number of transferred embryos among the three groups, while the number of retrieved oocytes, available embryos, and high-quality embryos were the lowest in the ANA+/anti-dsDNA+ group. Either difference of comparison of retrieved oocytes between the ANA+/anti-dsDNA+ group and the ANA+/anti-dsDNA− group (*P* < 0.001) or difference of comparison of retrieved oocytes between the ANA+/anti-dsDNA+ group and the ANA−/anti-dsDNA− group (*P* < 0.001) was statistically significant. There was no difference in retrieved oocytes between the ANA+/anti-dsDNA− group and the ANA−/anti-dsDNA− group (*P* = 0.439), which was conformed with our previous study outcome. Significant differences were found in the number of available embryos and high-quality embryos between any two groups. Significant difference was also found in fertilization rate by partition of chi-squared test between any two groups. Although the difference of implantation rate between the ANA+/anti-dsDNA+ group and the ANA+/anti-dsDNA− group (*P* = 0.176) was not statistically significant, either difference between the ANA+/anti-dsDNA+ group and the ANA−/anti-dsDNA− group(*P* = 0.001) or difference between the ANA+/anti-dsDNA− group and the ANA−/anti-dsDNA− group (*P* = 0.008) was statistically significant. In addition, the differences of pregnancy rate between any two groups were statistically significant. The pregnancy rate was the highest in the ANA−/anti-dsDNA− group. The early miscarriage rate was not statistically analyzed because of the small sample size (Table 2).

### 3.3. HRT-FET Outcomes among the Three Groups

HRT-FET outcomes were the same with IVF-ET outcomes. Although the difference of implantation rate between the ANA+/anti-dsDNA+ group and the ANA+/anti-dsDNA− group (*P* = 0.535) was not statistically significant, either difference between the ANA+/anti-dsDNA+ group and the ANA−/anti-dsDNA− group(*P* = 0.008) or difference between the ANA+/anti-dsDNA− group and the ANA−/anti-dsDNA− group (*P* = 0.015) was statistically significant. In addition, the differences of pregnancy rate between any two groups were statistically significant. The pregnancy rate was the highest in the ANA−/anti-dsDNA− group. The early miscarriage rate was not statistically analyzed because of the small sample size (Table 3).

## 4. Discussion

Many studies have investigated the association of ANA with adverse reproductive events including infertility, miscarriage, and implantation failure [1–4]. In our previous clinical study, we found rates of mature oocyte, normal fertilization, cleavage, high-quality embryo, implantation, and pregnancy in the ANA+ group which were inferior to the ANA− group notably [20]. Then, we collected follicular fluid and discarded the third-day embryos and found ANA existed in follicular fluid and human embryos using ELISA and immunofluorescence methods. The level of ANA in serum and follicular fluid have positive correlation. In addition, ANA positive in serum or follicular fluid was a risk factor for ANA positive in embryo. Patients detected ANA positive in discarded embryos achieved lower high-quality embryos and implantation rate, which may be a remainder that ANA also impaired high-quality transplanted embryos morphologically [21].

Antinuclear antibodies (ANAs) were a large group of autoantibodies targeting the entire cell including DNAs, RNAs, proteins, and/or their complexes. Anti-dsDNA antibody, one member of ANA, and serological marker for diagnosis of systemic lupus erythematosus could be eluted from the kidneys of patients with active nephritis, which suggested that the antibody might be important in induction of tissue damage. Anti-DNA and anti-RNP antibodies could actually enter living cells and interact with their intracellular target which was firstly reported by Alarcon-Segovia and colleagues in 1978 [9]. Many subsequent studies have confirmed this observation [10–13]. Following administration of anti-dsDNA antibody to nonautoimmune mice, anti-dsDNA antibody was detected in the nuclei of multiple cell types such as renal tubular cells, hepatocytes, neuronal cells, fibroblasts, and mononuclear cells. In the kidney, this was associated with glomerular hypercellularity and proteinuria. Nuclear localization was present after injection of F(ab) fragments of these anti-DNA antibodies, which indicated that localization occurred through the antigen binding region of the molecule and was FcR independent. Clinical symptoms of lupus nephritis appeared after injection of human or murine anti-dsDNA antibody into normal mice. Some studies demonstrated that anti-dsDNA could induce cell apoptosis after penetrated into cells [16–20]. Other studies also showed that intracellular penetration by anti-dsDNA antibody could upregulate mRNA expression of cytokines such as IL-1, IL-6, IL-8, and TGF-*β* [22, 23].

Apoptosis, named programmed cell death, is essential to embryonic development, homeostasis, surveillance, and elimination of pathological changes. Its main features are chromosome condensation, chromosome breakage, reduced cell volume, and apoptosis body. The damage of DNA degradation by endonuclease is irreversible in the process of apoptosis [24]. Apoptosis is important for the development of oocyte and preimplantation embryo under physiological circumstances. Arrested development and death would be occurred if apoptosis degree beyond physiological range. Low quality of embryos were associated with apoptosis [25, 26]. High level of DNA fragment in oocytes was related to low fertility by TUNEL detection [27]. Nowadays, the method of assessment of embryonic development potential was embryo morphological score in major reproductive medicine centers. But the predictive value of this method was limited and subjective. Recent study proved that regulatory mechanism of apoptosis was critical to preimplantation embryo potential and apoptosis degree of embryo was one key factor of reflection of embryo quality [28]. Anti-dsDNA antibody induced rat mesangial cell apoptosis without affecting p53 and Fas gene expression [15]. Human neutrophil cell apoptosis also took place as coculture with anti-dsDNA derived from lupus mice [16]. Increased cleavage of DNA caused by anti-dsDNA illustrated that anti-dsDNA induced cell apoptosis [17]. According to these lines of evidence, which offered indirect proof that anti-dsDNA antibody in patients with infertility might infiltrate into the oocytes and embryo and induce apoptosis. That may be the reason for poor reproductive outcomes. Without doubt, whether higher rate of apoptosis existed in oocytes and embryos from anti-dsDNA antibody positive patients and whether anti-dsDNA antibody could be detected directly in oocytes and embryos from anti-dsDNA antibody positive patients need to be verified in the future experiments.

## 5. Conclusion

To date, there is little information on the clinical significance of anti-dsDNA for improvement of artificial reproductive technology (ART) outcome. The present study analyzed IVF and HRT-TET outcomes among the ANA+/anti-dsDNA+ group, the ANA+/anti-dsDNA− group, and the ANA−/anti-dsDNA− group and found that the number of oocytes retrieved, available embryos, high-quality embryos, rates of fertilization, and clinical pregnancy were the lowest in the ANA+/anti-dsDNA+ group. All differences were significant. Although the early miscarriage rate was not statistically analyzed because of the small sample size, we found that all pregnant patients suffered from abortion in the ANA+/anti-dsDNA+ group and that anti-dsDNA could induce oocyte and embryo apoptosis through entering living cell may be the mechanism for poor reproductive outcomes. These results may provide new approaches to understand autoantibody involvement in reproductive outcomes for anti-dsDNA positive women suffering from IVF failure.

## Figures and Tables

**Table 1:** General characteristics among the three groups in the IVF cycle.

Variables	ANA+/anti-dsDNA+ group (*n* = 52)	ANA+/anti-dsDNA− group (*n* = 86)	ANA−/anti-dsDNA− group (*n* = 121)	*P* value
Age (y)	30.88 ± 4.29	31.41 ± 3.94	32.02 ± 3.73	0.262
BMI	20.93 ± 2.84	21.29 ± 2.79	21.40 ± 3.10	0.647
AFC	9.15 ± 1.35	9.12 ± 1.40	9.20 ± 1.16	0.825
Duration of infertility (y)	5.02 ± 2.79	4.85 ± 2.83	4.39 ± 3.02	0.093
Basal FSH (IU/l)	5.81 ± 1.51	6.02 ± 1.46	5.77 ± 1.44	0.312
Basal LH (IU/l)	3.98 ± 1.68	3.83 ± 1.62	4.03 ± 1.86	0.865
Basal E2 (pg/ml)	34.90 ± 12.21	34.36 ± 12.87	36.41 ± 12.01	0.416

*P* < 0.05 was considered to be statistically significant.

**Table 2:** Comparison of COS and IVF outcomes among the three groups.

Variables	ANA+/anti-dsDNA+ group (*n* = 52)	ANA+/anti-dsDNA− group (*n* = 86)	ANA−/anti-dsDNA− group (*n* = 121)	*P* value
Stimulation length (d)	11.06 ± 1.75	11.07 ± 1.70	10.80 ± 1.98	0.663
Total Gn dose (IU)	2404.17 ± 797.50	2400.24 ± 784.28	2337.06 ± 846.77	0.634
E2 level on HCG day (pg/ml)	3087.08 ± 927.41	3086.66 ± 1000.67	3183.55 ± 1086.38	0.866
Endometrial thickness on HCG day (mm)	10.50 ± 1.61	10.85 ± 1.80	10.67 ± 1.11	0.568
Number of retrieved oocytes	9.81 ± 1.19^A,C^	12.52 ± 3.50	12.89 ± 3.24	<0.001
Fertilization rate	51.0% (260/510) ^A,B,C^	57.9% (624/1077)	69.0% (1076/1560)	<0.001
Number of embryo transferred	2.04 ± 0.19	2.01 ± 0.11	2.02 ± 0.29	0.779
Available embryos	4.15 ± 0.85^A,B,C^	5.24 ± 1.23	5.81 ± 0.91	<0.001
High-quality embryos	2.13 ± 0.34^A,B,C^	2.99 ± 0.83	3.36 ± 0.79	<0.001
Implantation rate	9.4% (10/106) ^B,C^	15.0% (26/173)	25.8% (63/244)	<0.001
Clinical pregnancy rate	11.5% (6/52) ^A,B,C^	30.2% (26/86)	47.1% (57/121)	<0.001
Early miscarriage rate	100% (6/6)	38.5% (10/26)	22.8% (13/57)	—

^A,^ ^B,^ ^C^ *P* < 0.05 was considered to be statistically significant among the three groups. *P* < 0.0167 was considered to be statistically significant between any two groups. *P* < 0.0167 versus those of the groups ANA+/anti-dsDNA+ and ANA+/anti-dsDNA−. *P* < 0.0167 versus those of the groups ANA+/anti-dsDNA− and ANA−/anti-dsDNA−. *P* < 0.0167 versus those of the groups ANA+/anti-dsDNA+ and ANA−/anti-dsDNA−.

**Table 3:** Basal characteristics and HRT-FET outcomes among the three groups.

Variables	ANA+/anti-dsDNA+ group	ANA+/anti-dsDNA− group	ANA−/anti-dsDNA− group	*P*
Patients	32	48	56	—
Age (yrs)	30.78 ± 4.21	31.42 ± 3.79	32.43 ± 3.86	0.098
BMI	20.32 ± 2.38	21.15 ± 2.98	21.71 ± 3.54	0.176
Duration of infertility (yrs)	4.56 ± 1.64	4.38 ± 2.16	4.73 ± 0.92	0.093
bFSH (IU/l)	5.94 ± 1.68	5.95 ± 1.23	5.74 ± 1.70	0.546
bLH (IU/l)	3.98 ± 1.87	4.51 ± 1.72	3.85 ± 1.59	0.099
bE2 (pg/ml)	36.19 ± 12.62	32.65 ± 10.57	36.71 ± 12.29	0.186
Endometrial thickness on progesterone day (mm)	10.75 ± 1.65	10.65 ± 1.47	10.93 ± 1.02	0.482
E2 level on progesterone day (pg/ml)	160.66 ± 33.42	163.79 ± 29.06	158.70 ± 24.57	0.791
Embryo transferred	2.00 ± 0.25	2.04 ± 0.46	2.11 ± 0.31	0.382
Implantation rate (%)	10.9% (7/64)^B,C^	14.3% (14/98)	28.0% (33/118)	0.006
Clinical pregnancy rate (%)	9.4% (3/32)^A,B,C^	33.3% (16/48)	57.1% (32/56)	<0.001
Early miscarriage rate (%)	100% (3/3)	37.5% (6/16)	21.9% (7/32)	—

^A,^ ^B,^ ^C^ *P* < 0.05 was considered to be statistically significant among the three groups. *P* < 0.0167 was considered to be statistically significant between any two groups. *P* < 0.0167 versus those of the groups ANA+/anti-dsDNA+ and ANA+/anti-dsDNA−. *P* < 0.0167 versus those of the groups ANA+/anti-dsDNA− and ANA−/anti-dsDNA−. *P* < 0.0167 versus those of the groups ANA+/anti-dsDNA+ and ANA−/anti-dsDNA−.
